# A Study of the Effect of the Fringe Fields on the Electrostatic Force in Vertical Comb Drives

**DOI:** 10.3390/s141120149

**Published:** 2014-10-27

**Authors:** Else Gallagher, Walied Moussa

**Affiliations:** Department of Mechanical Engineering, University of Alberta, 4-9 Mechanical Engineering Building, Edmonton, AB T6G 2G8, Canada; E-Mail: else@ualberta.ca

**Keywords:** fringe electrostatic fields, vertical comb drives, net electrostatic force

## Abstract

The equation that describes the relationship between the applied voltage and the resulting electrostatic force within comb drives is often used to assist in choosing the dimensions for their design. This paper re-examines how some of these dimensions—particularly the cross-sectional dimensions of the comb teeth—affect this relationship in vertical comb drives. The electrostatic forces in several vertical comb drives fabricated for this study were measured and compared to predictions made with four different mathematical models in order to explore the amount of complexity required within a model to accurately predict the electrostatic forces in the comb drives.

## 1. Introduction

**Figure 1.**
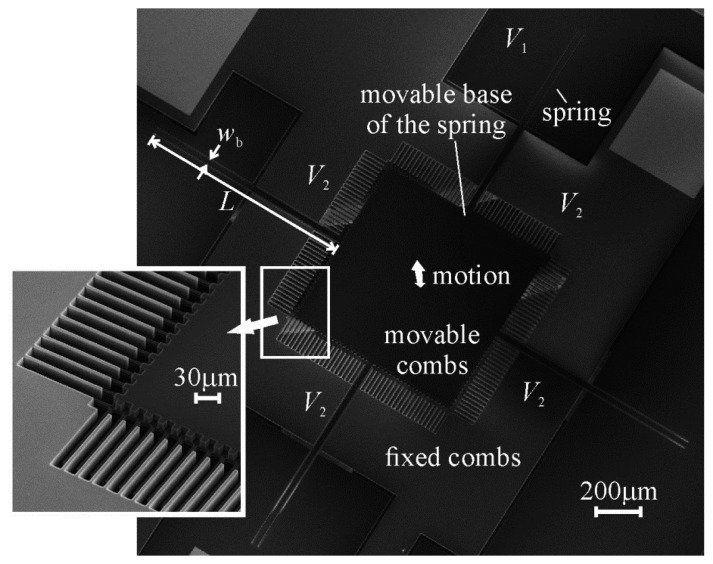
A vertical comb drive. Its components have been etched from the device layer of a silicon-on-insulator (SOI) wafer. The part of the handle wafer beneath its movable components has been removed. The thickness of its spring beams, *t*_b_, is equal to the thickness of the device layer.

Comb drives, such as the one shown in Figure 1, are electrostatic components of microelectromechanical systems (MEMS) that are comprised of arrays of parallel plates arranged into opposing comb pairs, where one comb in each pair is rigidly fixed to the substrate (the fixed comb), and the other comb is fixed to the substrate through a spring structure (the movable comb). If connected to a circuit that can measure the change in capacitance produced by the variance in overlapping area between their fixed and movable comb teeth as an external force is applied to the movable combs, comb drives can be used as sensors—such as accelerometers [1–3].

If a voltage difference is applied between their fixed and movable combs, comb drives can also be used as actuators, as the electrostatic forces generated between the combs attract the movable combs towards the fixed combs. The research presented here focuses on comb drives that have their plates (or teeth) staggered such that each movable tooth is laterally equidistant from two fixed teeth. In this case, the net electrostatic force on each movable tooth pulls it in between the fixed teeth—parallel to the fixed teeth. When the voltage difference is removed the springs restore the movable combs to their original positions.

Comb drives are often used as switches and micropositioners, controlling the position of MEMS components such as mirrors and microlenses [4–6]. They can also be used as force-compensation mechanisms, in, for instance, interfacial force microscopes. With a probe attached to their movable combs, a feedback circuit can apply a voltage difference between their fixed and movable combs so that the electrostatic force generated between them balances the interfacial forces felt by the movable combs as the probe interacts with a sample surface. The magnitudes of the interfacial forces would then be inferred from the magnitudes of the voltages used to balance them.

The advantages of using a comb drive as a force-compensation mechanism are that it can be made out of common conductive and insulating materials, and that its electrodes can be automatically aligned with each other during their manufacture. The advantage of using a vertical comb drive as a force-compensation mechanism in an interfacial force microscope is that the springs attached to its movable combs can be fabricated with a lower spring constant more easily than those designed to be compliant in a direction parallel to the surface of the substrate they are machined on [7]. The weaker the springs, the smaller the force that is required on the probe for it to reach the displacement prerequisite to activate the feedback control circuit. The vertically-offset combs of vertical comb drives also have the potential to apply electrostatic forces on the movable combs in both the upwards and downwards directions, allowing them to compensate for both attractive and repulsive interfacial forces acting on the attached probe.

This paper re-examines the relationship between the voltage applied between the fixed and movable combs of a vertical comb drive and the electrostatic force generated between them, as this relationship is often used to assist in choosing the dimensions of a comb drive's teeth. The basic case where the opposing teeth maintain the same relative height along their length, in the absence of a ground plane, is considered. Two-dimensional finite element models of the cross-sections of the teeth are studied in an attempt to add the effect of their width to a previously published force-voltage equation [8,9] that includes a first approximation of the effect of the fringe fields around the tops and bottoms of the teeth. The result is compared to electrostatic forces measured in several vertical comb drive prototypes.

## 2. Derivation of Basic Electrostatic Force Equation

The fixed and movable combs of a comb drive, being isolated conductors, form a capacitor when they are charged by the voltage difference applied between them. The fixed and movable combs have equal and opposite charges of +*q*_e_ and −*q*_e_, although the total charge between them is considered to be *q*_e_. The capacitance, *C*, of a capacitor is the proportionality constant that relates the charge on its opposing plates to the voltage difference, *V* (as in Equation (1)), and it is dependant only on the physical geometry of the plates [10]:

(1) $$q_{\text{e}}=CV$$

**Figure 2.**
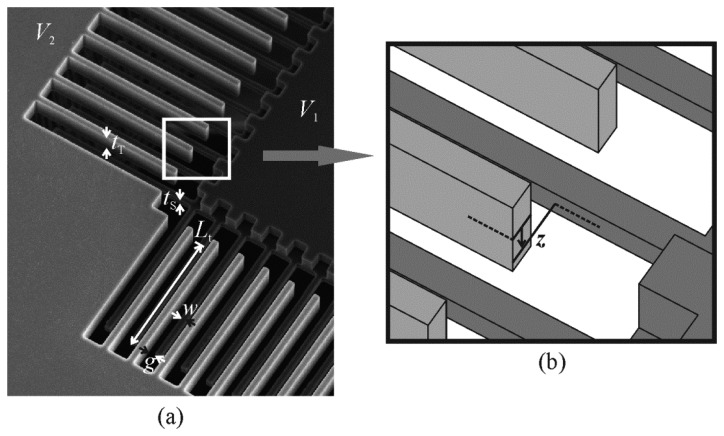
Some of the dimensions of the comb teeth that are expected to affect the electrostatic forces generated between them. (**b**) is an enlarged view of the area indicated in (**a**). It should be noted that “*t*_S_” refers to the thickness of the shorter teeth and “*t*_T_” to the thickness of the taller teeth, regardless of which are the fixed teeth and which are the movable teeth.

The physical geometry of the opposing combs in a comb drive is usually considered to consist of a number of overlapping parallel plates, that are assumed to be large enough and close enough together that the electric fields between them are uniform and confined to their overlapping regions (that is, the bending of the electric fields around the corners of the comb teeth can be ignored) [10]. The expression most often used to describe the capacitance between parallel plates is that of Equation (2), where ε is the permittivity of the medium between the plates, *g* is the lateral gap between the plates (shown in Figure 2), and *A*_c_ is their overlapping area:

(2) $$C=\frac{\varepsilon A_{\text{c}}}{g}$$

Summing the amount of work done to charge the combs against an increasing voltage difference, and using the relationship between the charge and voltage difference given in Equation (1), the energy stored in a capacitor, *U*, no matter its geometry, can be described as [10]:

(3) $$U=\frac{1}{2}CV^{2}$$

The net electrostatic force exerted on the movable combs of a comb drive, ***F***_e_, is typically found by considering the change in the electric potential energy between the comb teeth as they slide past each other. Taking the derivative of the energy with respect to the displacement of the movable combs, ***z***, gives Equation (4) for the portion of ***z*** where the teeth are partially overlapping, and zero everywhere else. The electrostatic force acts on the movable combs to pull them towards positions of increased capacitance. The two other parameters that represent the overlapping area of the opposing combs are the total number of movable teeth, *n* (assuming there are enough fixed teeth that they exert electrostatic forces on both sides of each movable tooth), and the overlapping length of the teeth, *L*_t_:

(4) $${\text{F}}_{\text{e}}=\frac{\partial U}{\partial \text{z}}=\frac{1}{2}\frac{\partial C}{\partial \text{z}}V^{2}=\frac{\varepsilon nL_{\text{t}}}{g}V^{2}$$

Equation (4) is the first estimate of the relationship between the electrostatic force, ***F***_e_, and the voltage applied between the fixed and movable combs of a comb drive, *V*. It is often used to assist in deciding what the gap between the teeth should be, how long the teeth should be, and how many teeth there should be in a particular comb drive design.

## 3. First Estimate of the Effect of the Fringe Fields on the Electrostatic Force

Fabrication techniques for micromachines such as comb drives tend to be limited to producing structures that are relatively thin in the direction perpendicular to the surface of the substrate they are machined on. Hence comb teeth are usually made to be thin, and long in a direction parallel to their substrate. If their fixed and movable combs are offset from each other parallel to the substrate surface (as in a lateral comb drive), there is a relatively long range over which the movable combs can displace before their ends approach the ends of the fixed teeth and restrict the overlapping region where the electric field between them can be considered to be uniform, and the electrostatic force can be described by Equation (4) [11–13]. On the other hand, if the fixed and movable combs are offset from each other perpendicular to the substrate (as in a vertical comb drive), there is a relatively short range over which the necessary amount of overlap can be maintained.

The failure of Equation (4) to include the fringe fields around the comb teeth comes from the failure of Equation (2) to include the fringe fields around the comb teeth. A method involving Schwartz–Christoffel transformations [14] has been heralded as providing an exact calculation of the capacitance between two-dimensional representations of aligned parallel plates [15,16]. It finds relations that transform the co-ordinates of the plates, being boundaries of the electric field (and between which the electric field lines are unknown curves), into co-ordinates in a new plane where the entire electric field is contained directly between the plates (between which the field lines are uniformly-spaced straight lines). The capacitance between the idealized plates can then be calculated with Equation (2), using the determined transformation relations to find the dimensions of the idealized plates from the dimensions of the real plates.

Unfortunately, the Schwartz–Christoffel transformation method for calculating capacitance cannot be expressed as a single equation, so its derivative cannot simply be taken and used to calculate the electrostatic force between the plates as in Equation (4). Much of the calculation process must be done numerically, and even further numerical steps are needed if the widths of the plates are to be included or plates of differing thicknesses are being considered [15]. The method is also limited to the analysis of parallel plates that have their centres aligned with each other. The centres of opposing comb teeth need to be offset from each other if there is to be a net force between them.

**Figure 3.**
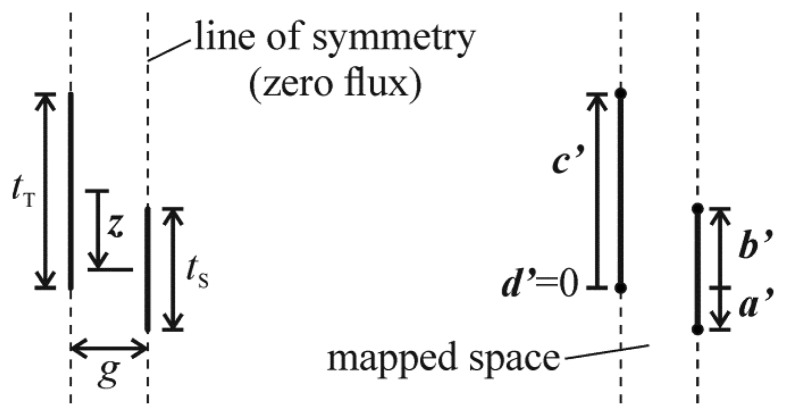
Two different representations of the two-dimensional model of the space between opposing teeth of negligible width.

One attempt that has been made [8,9] at including the fringe electric fields in the calculation of the electrostatic force in vertical comb drives involves the conformal mapping of a two-dimensional model of half of a unit comb drive. The model is comprised of the space between zero-width cross-sections of the opposing halves of one fixed comb tooth and one movable comb tooth, as in those models shown in Figure 3. The end result of this work is effectively a function, ***f***, that is appended to Equation (4), that describes the effect of the fringe field on the electrostatic force. It predicts a changing electrostatic force over ***z***, and is able to predict a force in the ranges of ***z*** where the comb teeth do not partially overlap. The form of ***f*** that was derived, ***f***_1_, is shown in Equation (5) and is based on the positions of the tops and bottoms of the comb teeth (***a′***, ***b′***, ***c′***, and ***d′***—relative to a scale downwards from ***d′***), or, the relative position of the teeth, the thicknesses of the teeth, and the gap between them (***z***, *t*_S_, *t*_T_, and *g*):

(5) $$\begin{array}{ll} {\text{F}}_{\text{e}} & =\frac{\varepsilon nL_{\text{t}}}{g}V^{2}{\text{f}}_{1}\left(g,t_{\text{S}},t_{\text{T}},\text{z}\right)\\ & =\frac{\varepsilon nL_{\text{t}}}{g}V^{2}\cdot \frac{\pi^{2}\left(a\frac{b-d}{a-d}-b\frac{a-c}{b-c}\right)}{4\left(a-b\right){\left\{F\left[\delta \left(u=0\right),q\right]+F\left[\kappa \left(u=0\right),q\right]\right\}}^{2}} \end{array}$$

where:

(5a) $$q=\sqrt{\frac{\left(b-c\right)\left(a-d\right)}{\left(a-c\right)\left(b-d\right)}}$$

(5b) $$\delta =\sin^{-1}\sqrt{\frac{\left(b-d\right)\left(u-c\right)}{\left(b-c\right)\left(u-d\right)}}$$

(5c) $$\kappa =\sin^{-1}\sqrt{\frac{\left(a-c\right)\left(b-u\right)}{\left(b-c\right)\left(a-u\right)}}$$

and *F* is the incomplete elliptic function of the first kind—one representation of which is given in Equation (5d) [17]. It must be approximated numerically:

(5d) $$F\left(\varphi ,k\right)=\int_{0}^{\sin\varphi }\frac{dp}{\sqrt{\left(1-p^{2}\right)\left(1-k^{2}p^{2}\right)}}$$

The remaining variables—*a*, *b*, *c*, and *d*—can be related to the physical endpoints of the teeth through Equation (5e–h):

(5e) $$a={\text{e}}^{\pi a^{\prime }/g}={\text{e}}^{\pi \left(b^{\prime }+t_{\text{S}}\right)/g}$$

(5f) $$b={\text{e}}^{\pi b^{\prime }/g}$$

(5g) $$c=-{\text{e}}^{\pi c^{\prime }/g}=-{\text{e}}^{-\pi t_{\text{T}}/g}$$

(5h) $$d=-{\text{e}}^{\pi d^{\prime }/g}=-1$$

Finally, relating ***b′*** to ***z*** is trivial—the co-ordinate system chosen for the work presented here has its origin at the midpoint of the taller comb teeth, and increases as the midpoint of the shorter comb teeth travels (relative to the taller teeth) in the direction outwardly normal to the substrate surface, thus:

(5i) $${\text{b}}^{\prime }=-\text{z}-\left(\frac{t_{\text{T}}+t_{\text{S}}}{2}\right)$$

**Figure 4.**
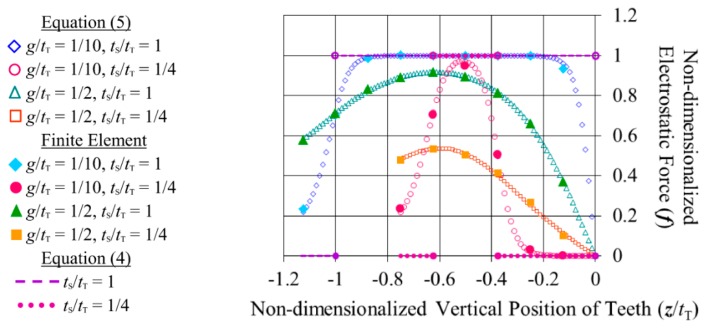
Different predictions of the fraction of the maximum electrostatic force that acts on the movable combs over their displacement.

Equation (5) is shown graphically in Figure 4 for the cases where *g*/*t*_T_ = 1/10 and 1/2, and where the fixed and movable tooth are the same thickness, and where one is a quarter of the thickness of the other. (These are values that the dimensions of typical microfabricated vertical comb teeth are expected to fall between.) The relative position of the teeth is varied from where the vertical midpoints of the teeth are aligned horizontally, to a little beyond where the teeth no longer overlap (for the *t*_S_/*t*_T_ = 1 case they no longer overlap at ***z***/*t*_T_ = −1, and for the *t*_S_/*t*_T_ = 1/4 case they no longer overlap at ***z***/*t*_T_ = −0.625). Since the attractive force on a tooth pulls it towards the vertical midpoints of its opposing teeth, the force on the tooth always acts in the opposite direction to its position vector (that is, if the positions of the teeth were varied around ***z***/*t*_T_ ≥ 0, the forces on the shorter tooth would be of the same magnitude but in the negative direction). In the figure, Equation (5) has been non-dimensionalized with respect to Equation (4) (which produces a straight line at a force of 1 while the teeth partially overlap). Not surprisingly, the figure shows that a larger gap between the teeth lowers the force between them, and also lowers the rate of change of the force as the teeth come to completely overlap, and as they move to not overlap at all. Having one tooth shorter than the other decreases the maximum force and narrows the distance over which the force acts, as there is a longer range of travel where the teeth completely overlap.

## 4. Addition of Tooth Width to Electrostatic Force Calculations

### 4.1. Verification of Finite Element Models

The derivation of Equation (5) assumes that the comb teeth are narrow enough that their width does not affect the electrostatic forces between them. Having wider teeth is advantageous during fabrication process development, however, as they are more likely to survive being etched if the amount of undercut of the masking layer to expect is unknown, and they are less likely to break during their release from the substrate. Hence finite element models of the air around the cross-section of a pair of comb teeth were developed in the program COMSOL 3.5a to calculate the capacitances of different tooth configurations. Initially, the models were made with zero-width teeth so as to compare their results with those in the literature.

A finite element model with a unit potential difference applied between an example pair of teeth (that were separated by a gap equal to half their thickness) calculated the same capacitance of 2.89 × 10^−13^ F/cm as the Schwartz-Christoffel method [14]. In subsequent models, to represent pairs of teeth that are a part of an array of teeth, only the space around half of each tooth was included, and symmetric conditions were applied to the boundaries extending from the teeth along their vertical axes. These models were used to calculate a number of capacitances for different relative vertical positions of the teeth. The differences between the capacitances were taken, divided by the respective changes in position, and multiplied by “½*V*^2^”, as in Equation (4). The resulting electrostatic forces have been plotted against those predicted by Equation (5) in Figure 4. On average, they deviate from those predicted by Equation (5) by 0.6% of the maximum force predicted by Equation (4), with a maximum of 2.8%.

### 4.2. Addition of Width to Plate Model

**Figure 5.**
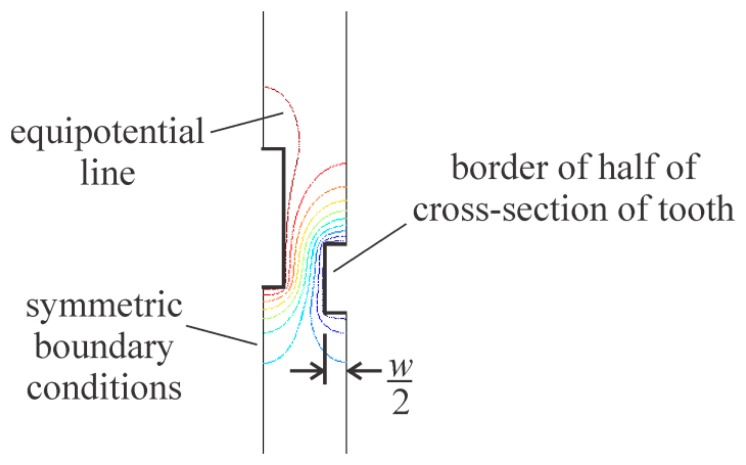
One of the finite element models that includes the width of the comb teeth.

The width of the comb teeth was added to the finite element models by extending the discretized space horizontally to half the width of both teeth, as shown in Figure 5. The precise dimensions chosen for the models were those measured from a vertical comb drive that was fabricated for this study (the one shown in Figure 1).

The comb drive was made in the device layer of a silicon-on-insulator (SOI) wafer. The vertical offset between its teeth was created by etching down the tops of its movable combs. The handle wafer was etched away from beneath its movable combs and springs, and its movable components were released from the substrate by etching away the buried oxide beneath them [7].

**Figure 6.**
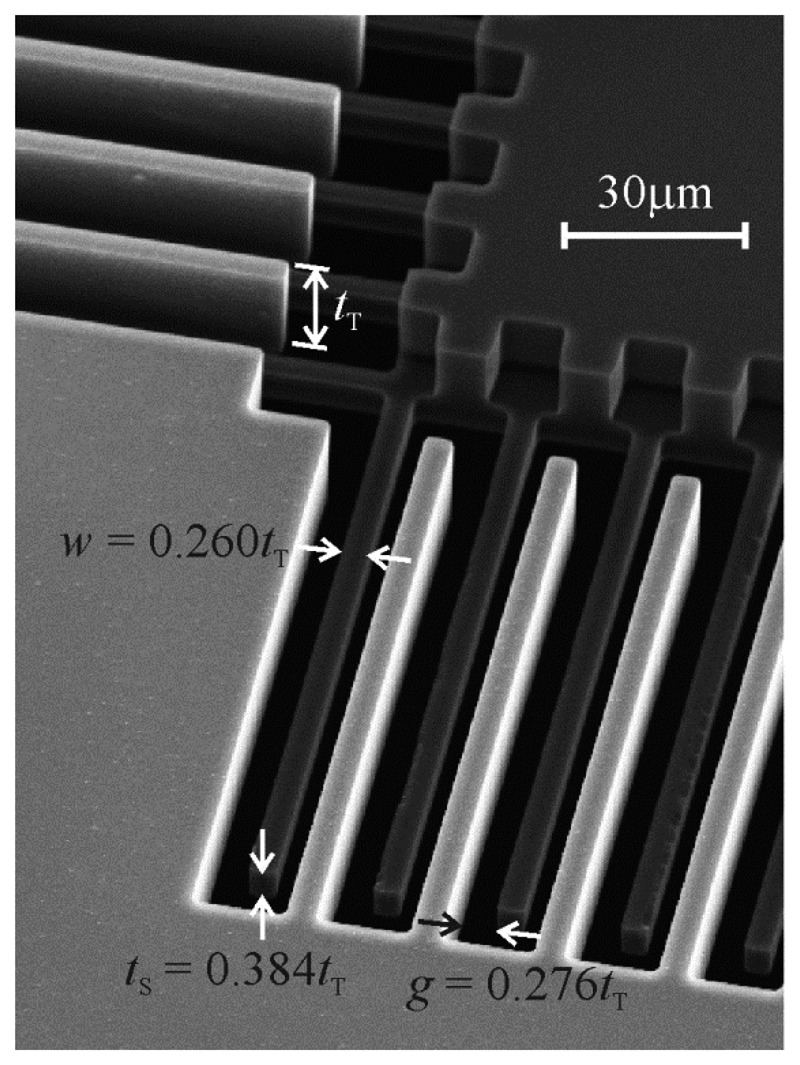
The relative tooth dimensions of one of the vertical comb drives fabricated for this study.

The comb drive has 160 movable teeth, and the four beams that extend in opposing directions from the movable combs act as its springs. The overlapping length of the teeth was estimated from pictures taken in a scanning electron microscope to be 99.24 μm, and the relative width of, gap between, and thickness of the teeth are those shown in Figure 6 of 0.260*t*_T_, 0.276*t*_T_, and 0.384*t*_T_, respectively, although the thickness of the shorter teeth was determined by subtracting the depth of the etch of their tops, measured with a Zygo NewView optical profilometer, from the thickness of the taller teeth.

**Figure 7.**
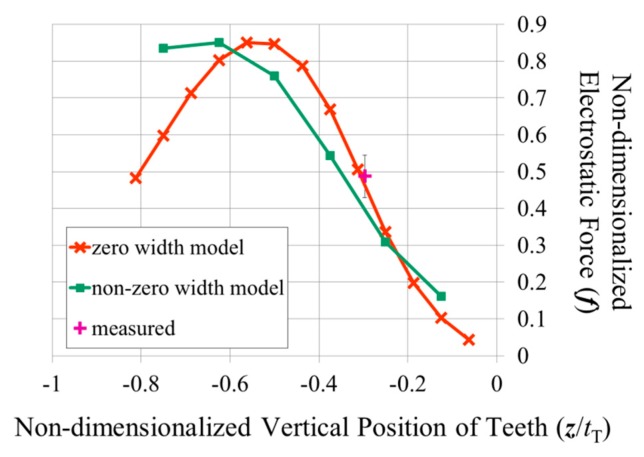
A comparison of two different predictions of the electrostatic force to that measured in the vertical comb drive shown in Figures 1 and 6 that has tooth dimensions of *w*/*t*_T_ = 0.260, *g*/*t*_T_ = 0.276, and *t*_S_/*t*_T_ = 0.384. The electrostatic forces have been non-dimensionalized with respect to Equation (4).

Figure 7 shows the electrostatic forces predicted for this comb drive using Equation (5) and using the capacitances obtained from the finite element models. The largest difference between the two predictions occurs when the comb teeth no longer overlap in the vertical direction. When the top of one tooth is facing the bottom of the other, including the width of the teeth in the calculation increases the area over which the electric charge can accumulate, resulting in a higher predicted electrostatic force.

## 5. Electrostatic Force Measurements

20 V was applied to the fixed combs of the comb drive using electrical probes and an Agilent E3647A DC power supply. The movable combs and the handle wafer were grounded. The optical profilometer was used to measure the relative heights of the opposing combs while the voltage was applied, as well as the step height between the movable bases of the springs and a fixed reference surface (in an area of the device layer over a remaining portion of the handle wafer) before and after the voltage was applied. 20 V was found to raise the ends of the springs by an average of 44 nm.

**Figure 8.**
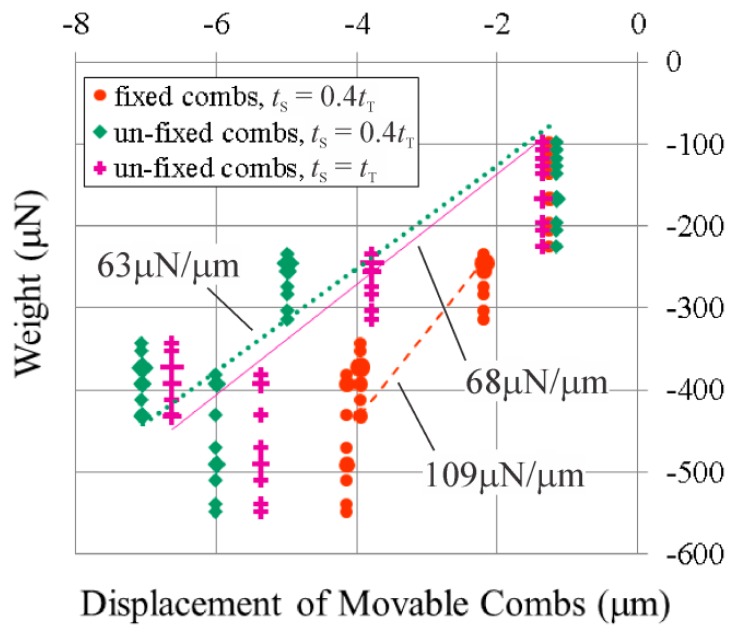
The measured spring constants of the three vertical comb drive designs. A few of the data points have been made larger to indicate that those values of the weights were recorded two or three times.

This displacement was used to calculate the electrostatic force generated by the 20 V by multiplying it by the measured stiffness of the springs, as the electrostatic force would be equal to the restoring force provided by the springs after the voltage had drawn the movable combs up to their new position. The stiffness of the springs was determined by using the optical profilometer to measure the displacement of the movable combs while a series of four weights were applied to their centre. The weights were applied using a lever mechanism [7]; the amount of weight the tip of the lever applied was measured by placing it on an Acculab Sartorius VIC-303 scale. 10 measurements of each weight were taken and multiplied by 9.81 m/s^2^ to convert them to units of force, and plotted against the resulting displacement of the movable combs in Figure 8. It was presumed that smaller displacements were generally produced with less weight, so the spring constant was calculated from linear regression performed between the values of the measured weights in the parts of the ranges of the measured weights that overlapped. The ranges of error for the spring constants were calculated from lines fit to the measured weights above and below the lines calculated for the spring constants.

The spring constant of the comb drive that underwent 44 nm of displacement at 20 V was thus measured to be 109 μN/μm (96 μN/μm–122 μN/μm). This is reasonably close to the 113 μN/μm calculated using Equation (6):

(6) $$k_{z+}=\frac{4Ew_{\text{b}}t_{\text{b}}^{3}}{L^{3}}$$

where *E* is the modulus of elasticity of the silicon, *L* is the length of the spring beams (as shown in Figure 1), *w*_b_ is the width of the spring beams, and *t*_b_ is their thickness. The spring beams were oriented along the <100> directions of the (100) device layer of the wafer in order to minimize their modulus of elasticity, which was taken to be 130.2 GPa [18]. The length, width, and thickness of the beams were measured in a scanning electron microscope to be 1065 μm, 30.34 μm, and 20.5 μm, respectively. The measured spring constant was also reasonably close to the 107 μN/μm calculated from the displacement of a finite element model of the movable combs when different weights were applied to their centre. Using the measured spring constant, the voltage difference was estimated to have generated 4.8 μN (4.3 μN–5.4 μN) of electrostatic force between the opposing combs. This measured electrostatic force has been non-dimensionalized with respect to Equation (4) and plotted in Figure 7 against Equation (5) and the forces predicted with the non-zero width plate model. The measured force seems to coincide with the predicted forces rather well, although there is not a large difference between the two predictions at this relative height of the teeth.

**Figure 9.**
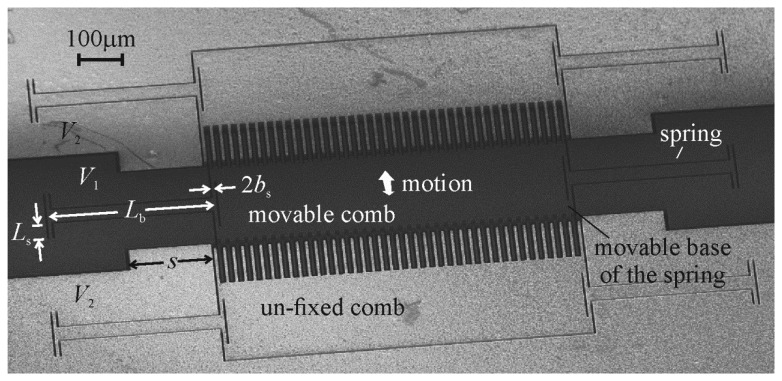
The vertical comb drive design that has its “fixed” combs also attached to springs so that they can be pushed down with the electrical probes to allow the electrostatic forces between the fixed and movable teeth to be measured over a larger range of vertical offsets. The thickness of the narrow spring beams, 2*a*_s_, is equal to the thickness of the device layer the comb drive is etched from.

To increase the range of relative heights of the opposing comb teeth over which the electrostatic force could be measured, another set of vertical comb drives were designed that had their “fixed” combs also attached to springs, so that the probes used to apply the voltages to the fixed combs could also be used to push them downwards to create different vertical offsets between them and the movable combs. No adjustments to the fabrication process for the comb drives were needed, only the outlines of the comb drives that were etched through the device layer of the SOI wafer were changed. As can be seen in Figure 9, the springs were re-designed so that the two “un-fixed” combs could fit on either side of the movable combs. The vertical compliance of the new springs comes from the twisting of the smaller spring beams [5], which allows the larger beam that connects them to be wider—which helps resist the tilting of the movable combs about the axis of the wide beams.

The displacements of the movable combs in the new comb drives were measured in the same way as the displacement of the movable combs in the first comb drive; however, the vertical offset between the teeth of the new comb drives was set by the height at which the Quater XYZ micropositioners held the probes that applied the voltage to the un-fixed combs. Before voltages were applied to the combs, the optical profilometer was used to ensure that the two un-fixed combs were pushed down evenly. The difference in their height was kept below 500 nm, or approximately 0.024*t*_T_. Their relative position to the movable combs when the voltage was applied was then taken as an average between them.

The comb drive shown in Figure 9 has 80 movable teeth. The overlapping length of the teeth was measured to be 98.26 μm, and the relative width of, gap between, and thickness of the teeth are 0.241*t*_T_, 0.296*t*_T_, and 0.365*t*_T_, respectively. The stiffness of its springs was measured with the lever mechanism to be 63 μN/μm (54 μN/μm–79 μN/μm). In this case, because not all of the ranges of the measured weights fell on the same force-displacement line, it was presumed that the process of placing the tip of the lever in the centre of the movable combs could produce weights outside of the range measured with the scale, so the measured spring constants were calculated from linear regression performed between the endpoints of the non-overlapping ranges in order to minimize the distance outside of the ranges the applied weights were estimated to be. The range of error for the spring constant was still calculated from lines fit to the measured weights above and below the line calculated for the spring constant. The measured spring stiffness is close to the 71 μN/μm calculated from a finite element model of this spring design, and the 53 μN/μm calculated using Equation (7):

(7) $$k_{z\text{I}}=\frac{8Ga_{\text{s}}b_{\text{s}}^{\text{3}}}{L_{\text{s}}L_{\text{b}}^{2}}\left[\frac{16}{3}-3.36\frac{b_{\text{s}}}{a_{\text{s}}}\left(1-\frac{b_{\text{s}}^{4}}{12a_{\text{s}}^{4}}\right)\right]$$

where *G* is the bulk shear modulus of the silicon, *L*_b_ is the length of the wide rigid beams (measured between the centre points of the narrow torsion beams, shown in Figure 9), *L*_s_ is the length of the torsion beams, and 2*a*_s_ and 2*b*_s_ are the cross-sectional dimensions of the torsion beams (where *a*_s_ > *b*_s_). 2*a*_s_, 2*b*_s_, and *L*_s_ were measured in a scanning electron microscope to be 20.5 μm, 5.931 μm, and 58.74 μm, respectively, while *L*_b_ was assumed to retain the designed value of 400 μm, as this dimension should not be affected by any undercutting in the etch of the silicon. The bulk shear modulus of the torsion beams was calculated according to the aspect ratio of their cross-sectional dimensions and their orientation within the silicon [18]. They were oriented at 45° to the <100> direction in the device layer of the wafer so as to minimize the bulk shear modulus, which was calculated to be 53 GPa.

**Figure 10.**
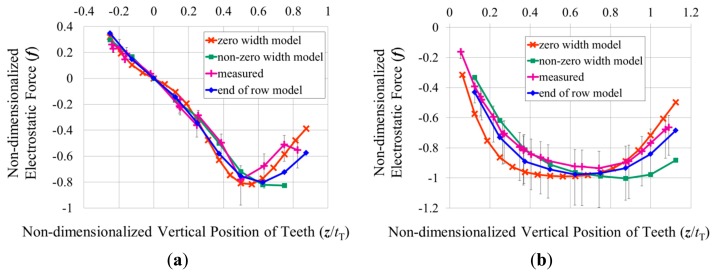
Comparisons of three different predictions of the electrostatic forces to those measured in (**a**) a vertical comb drive that has shorter movable teeth than fixed teeth (with tooth dimensions of *w*/*t*_T_ = 0.241, *g*/*t*_T_ = 0.296, and *t*_S_/*t*_T_ = 0.365) and (**b**) a vertical comb drive that has fixed and movable teeth of the same size (*w*/*t*_T_ = 0.234, *g*/*t*_T_ = 0.300, and *t*_S_/*t*_T_ = 1). The electrostatic forces have been non-dimensionalized with respect to Equation (4).

35 V was applied between the opposing combs of the comb drive at each position its un-fixed combs were held at. The resulting forces that were measured using the optical profilometer are shown in Figure 10a. In this comb drive design the movable teeth are the shorter teeth, so they were pulled up towards the centres of the un-fixed teeth when the un-fixed teeth were held only slightly below their fabricated positions, and the movable teeth were pulled downwards when the un-fixed teeth were pushed down so that their centres were below those of the movable teeth.

The method of varying the relative heights of the combs by pushing down “un-fixed” combs also allowed forces to be measured in a comb drive that has opposing comb teeth of the same thickness. This final comb drive prototype also has 80 movable teeth, which overlap the un-fixed teeth by 99.98 μm, while the relative width of the teeth is 0.234*t*_T_, and the gap between them is 0.300*t*_T_. The stiffness of its springs was measured with the lever mechanism to be 68 μN/μm (60 μN/μm–87 μN/μm), which corresponds well to the 69 μN/μm calculated from a finite element model of its springs, although it corresponds a little less so to the 50 μN/μm calculated from Equation (7), where 2*a*_s_, 2*b*_s_, and *L*_s_ were measured to be 20.5 μm, 5.789 μm, and 57.02 μm, respectively, while *L*_b_ was again assumed to be 400 μm, and *G* was calculated to be 52 GPa. 35 V was applied between the opposing combs of the comb drives at each position its un-fixed combs were held at. The resulting forces that were measured using the optical profilometer are shown in Figure 10b. Since in this comb drive design all of the teeth are the same thickness, the force on the movable teeth was plotted, and again, this force acted in the negative direction as the movable teeth remained above the un-fixed teeth as the un-fixed teeth were pushed down by the probes.

## 6. Addition of End of Row to Model

The magnitudes of the measured electrostatic forces seem to lie between those predicted with the finite element models of the unit comb drives and the zero-width approximation of Equation (5). This is particularly noticeable for the comb drive that had fixed and movable teeth of the same thickness, as the two different predictions are very close to each other for the other two comb drives that had movable teeth that were around four tenths of the thickness of their fixed teeth.

**Figure 11.**
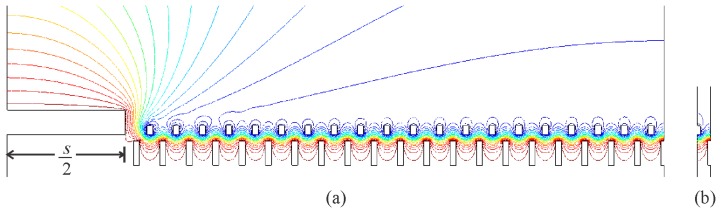
(**a**) One of the models of a row of teeth with a portion of the device layer at the end. The equipotential lines show the electric field being pulled away from the tops of the shorter teeth, lessening the effect of the width of the teeth on the electrostatic force between them; (**b**) The corresponding unit comb drive.

The 2-D finite element models of the cross-sections of the comb teeth were expanded as shown in Figure 11 to include half of a row of teeth (that is, 20 movable teeth and their counterparts) and the cross-section of the area of the device layer at the end of the row of teeth—to a length of half of the dimension *s*, shown in Figure 9.

Symmetric boundary conditions were applied to the two ends of the models, and capacitances were calculated at different relative heights of the teeth as before. The resulting electrostatic forces were non-dimensionalized with respect to Equation (4), and plotted in Figure 10. This final prediction of the electrostatic forces seems to match well with those measured from the fabricated comb drives. As can be seen from the equipotential lines in Figure 11, the portion of the device layer at the end of the row of teeth appears to be drawing the electric field away from the tops and bottoms of the teeth. Hence for the ranges of relative heights of the teeth where the width of the teeth is expected to affect the electrostatic force between them, including a portion of the electrodes around the teeth in the model seems necessary to determine how much the width of the teeth will affect the electrostatic force.

## 7. Discussion of the Significance of Including the Fringe Fields in the Electrostatic Force Calculation

Besides allowing an estimate to be made of the complexity of the mathematical model required to accurately predict the electrostatic forces within vertical comb drives, the collection of measured electrostatic forces was valuable in another way: it indicated that the first comb drive fabricated for this study will only generate about half the electrostatic force for any given voltage than that predicted by Equation (4)—the equation traditionally used to determine the dimensions of the comb teeth required for a particular force-voltage relationship. For example, if the comb drive shown in Figure 1 were used as a force-compensation mechanism, and it were possible to detect a change in the compensation voltage as small as 10 mV, theoretically, once an external force had pushed the movable combs to the displacement detection limit of the system, the comb drive could compensate for changes in the force on the movable combs as small as 1 pN (or 5 nN, if, for instance, 19 V had already been applied to pull against 4 μN). These values were obtained by adding a factor of 0.5 to Equation (4). If the effect of the fringe fields were to be neglected, such a comb drive would be designed to have, for example, half the number of comb teeth that it needed to achieve these forces.

## 8. Conclusions

The equation that describes the relationship between the applied voltage and the resulting electrostatic force within comb drives is often used to assist in choosing the dimensions for their design. This paper re-examines how some of these dimensions—particularly the cross-sectional dimensions of the comb teeth—affect this relationship in vertical comb drives.

The electrostatic force is a function of how the capacitance between the teeth changes with respect to their relative vertical positions. Traditionally, a poor estimate of the capacitance, that does not include the fringe electric fields around the tops and bottoms of the comb teeth, has been used to calculate electrostatic forces. An analytical force-voltage relationship [8,9] was found in the literature that includes the fringe fields around the tops and bottoms of zero-width teeth, and 2-D finite element models were made of the cross-sections of pairs of teeth to determine the difference the inclusion of their width would make to the prediction of the electrostatic forces. The electrostatic forces predicted with both methods were compared to those measured in comb drives fabricated for this study.

The measured electrostatic forces seemed to lie between those predicted with the two different methods, but they matched well with those predicted with 2-D models that were extended to include the cross-sections of the rest of the teeth in the row, as well as a portion of the area of the comb drive at the end of the row. Hence for the ranges of relative heights of the teeth where the width of the teeth is expected to affect the electrostatic force between them, including a portion of the electrodes around the teeth in the model seems necessary to determine how much the width of the teeth will affect the electrostatic force—as the effect of the width is lessened by the areas around the combs drawing the electric fields away from the tops and bottoms of the teeth.
